# Initial safety report on the tolerability of modified FOLFOX6 as adjuvant therapy in patients with curatively resected stage II or III colon cancer (JFMC41-1001-C2: JOIN trial)

**DOI:** 10.1007/s00280-015-2757-0

**Published:** 2015-05-16

**Authors:** Masahito Kotaka, Takayuki Yoshino, Koji Oba, Katsunori Shinozaki, Tetsuo Touyama, Dai Manaka, Takanori Matsui, Kiyoshi Ishigure, Junichi Hasegawa, Keiji Inoue, Koichi Goto, Junichi Sakamoto, Shigetoyo Saji, Atsushi Ohtsu, Toshiaki Watanabe

**Affiliations:** Gastrointestinal Cancer Center, Sano Hospital, Kobe, Japan; Department of Gastroenterology and Gastrointestinal Oncology, National Cancer Center Hospital East, 6-5-1 Kashiwanoha, Kashiwa, Chiba 277-8577 Japan; Department of Biostatistics, School of Public Health, Graduate School of Medicine and Interfaculty Initiative in Information Studies, The University of Tokyo, Tokyo, Japan; Division of Clinical Oncology, Hiroshima Prefectural Hospital, Hiroshima, Japan; Department of Surgery, Nakagami Hospital, Okinawa, Japan; Department of Surgery, Gastrointestinal Center, Kyoto-Katsura Hospital, Kyoto, Japan; Department of Gastroenterological Surgery, Aichi Cancer Center Aichi Hospital, Okazaki, Japan; Department of Surgery, Konan Kosei Hospital, Konan, Japan; Department of Surgery, Osaka Rosai Hospital, Sakai, Japan; Department of Surgery, Nagasaki Harbor Medical Center City Hospital, Nagasaki, Japan; Division of Thoracic Oncology, National Cancer Center Hospital East, Kashiwa, Japan; Japanese Foundation for Multidisciplinary Treatment of Cancer, Tokyo, Japan; Exploratory Oncology Research and Clinical Trial Center, National Cancer Center, Tokyo, Japan; Department of Surgical Oncology, The University of Tokyo, Tokyo, Japan

**Keywords:** Colon cancer, mFOLFOX6, Adjuvant, Peripheral sensory neuropathy, Allergic reactions

## Abstract

**Purpose:**

Adjuvant FOLFOX is a widely accepted standard therapy for resected colon cancer. The incidence of grade 3–4 peripheral sensory neuropathy (PSN) was 12.4 and 5.7 % in the MOSAIC and Eastern MASCOT trials, while that of grade 3–4 allergic reactions (AR) was 2.9 and 3.1 %, respectively. The JFMC41-1001-C2 trial (JOIN trial) investigated the tolerability of modified FOLFOX6 (mFOLFOX6) in Japanese colon cancer patients.

**Methods:**

Twelve cycles of mFOLFOX6 were given to patients with the same eligibility criteria as in the MOSAIC study: stage II or III curatively resected colon cancer, performance status of 0–1, aged 20 years or older, starting mFOLFOX6 within 7 weeks of surgery, and adequate organ function. The primary endpoints were the incidence of PSN persisting for ≥8 days that interfered with daily activities and the incidence of grade 3–4 AR. The target sample size was 800.

**Results:**

From November 2010 to March 2012, 882 patients were enrolled at 198 institutions. Safety was analyzed in 828 patients with finalized data out of 848 patients receiving mFOLFOX6. The incidence of PSN persisting ≥8 days was 3.3 % [95 % confidence interval (CI) 2.2–4.7], while that of grade 3–4 AR was 1.7 % (95 % CI 0.9–2.8). The treatment completion rate was 67.0 %. The median total dosage of oxaliplatin was 811.1 mg/m^2^. The overall incidence of grade 3–4 PSN was 5.8 %. Interstitial pneumonitis occurred in one patient. There were no treatment-related deaths.

**Conclusions:**

Adjuvant mFOLFOX6 is tolerable for Japanese patients with colon cancer.

## Introduction

FOLFOX is the combination of 5-fluorouracil (5-FU) and *l*-leucovorin (*1*-LV) with oxaliplatin (L-OHP) and is recommended as one of the standard adjuvant chemotherapy regimens for patients with curatively resected colon cancer by the National Comprehensive Cancer Network (NCCN) guidelines [1]. In Japan, it is also recommended by the 2010 Guidelines for the Treatment of Colorectal Cancer of the Japanese Society for Cancer of the Colon and Rectum (JSCCR) [2]. Adjuvant FOLFOX is indicated for patients who have stage III colon cancer with lymph node metastasis, as well as for patients with high-risk stage II colon cancer who have risk factors for recurrence that are known to be associated with a relatively poor prognosis, such as T4 status, poorly differentiated histology, vascular invasion, ileus, <12 lymph nodes examined, and neural invasion [1].

The MOSAIC trial was a large-scale randomized controlled trial performed mainly in Europe that assessed the efficacy and safety of FOLFOX4 as adjuvant therapy [3, 4]. Significant improvement in disease-free survival (DFS) and overall survival (OS) was seen in the FOLFOX4 group compared with the LV5FU2 group (receiving *1*-LV modulated infusional 5-FU therapy). In addition, the NSABP C-08 trial confirmed that modified FOLFOX6 (mFOLFOX6) therapy was equivalent to FOLFOX4 therapy in terms of efficacy and safety [5, 6].

Either adjuvant FOLFOX4 or mFOLFOX6 is routinely given as 12 courses (2 weeks per course). However, continuation of treatment is often interrupted/discontinued by the development of severe peripheral sensory neuropathy (PSN) and allergic reactions/anaphylaxis (AR). In the MOSAIC trial, the incidence of grade ≥1 PSN and grade ≥1 AR due to FOLFOX4 therapy was 92.0 and 10.3 %, respectively, while grade ≥3 PSN and grade ≥3 AR had an incidence of 12.4 and 2.9 %, respectively [3, 4]. In the MASCOT trial, which was conducted to confirm the safety of FOLFOX4 therapy in Asian countries other than Japan (China, Hong Kong, South Korea, Taiwan, and Thailand), the incidence of grade ≥1 PSN and grade ≥1 AR was 86.2 and 25.2 %, respectively, while grade ≥3 reactions showed an incidence of 5.7 and 3.1 %, respectively: The incidence of grade ≥3 PSN and grade ≥1AR differed between the two trials [7].

In the NSABP C-08 trial, adjuvant mFOLFOX6 therapy caused grade ≥3 PSN and grade ≥3 AR at an incidence of 14.4 and 4.7 %, respectively [5], but to our knowledge, the incidence of grade ≥1 PSN and grade ≥1 AR has not been reported yet. In addition, there are no safety data for adjuvant mFOLFOX6 therapy in the Asian population. Therefore, we conducted the present study (JFMC41-1001-C2; JOIN trial) to confirm the tolerability of adjuvant mFOLFOX6 therapy in Japanese patients (UMIN ID: 000004443). Here, we report on the initial safety data from the JOIN trial.

## Patients and methods

### Eligibility criteria

Eligibility criteria for this study corresponded to those for the MOSAIC trial and were as follows: a pathological stage II (T3-4N0M0) or stage III (TanyN1-2M0) colon cancer [8] (including cancer of the rectosigmoid region); curative resection (curative level A) and no macroscopic and microscopic residual tumors; starting mFOLFOX6 therapy within 7 weeks after surgery and within 2 weeks after enrollment; an Eastern Cooperative Oncology Group (ECOG) performance status (PS) of 0–1; an age of 20 years or older; no prior chemotherapy, immunotherapy, or radiation therapy; adequate function of vital organs [neutrophil count ≥1500/mm^3^, platelet count ≥100,000/mm^3^, serum creatinine ≤1.25× institutional upper limit of normal (ULN)]; total bilirubin <2× ULN, aspartate aminotransferase and alanine aminotransferase <2× ULN, and carcinoembryonic antigen <10 ng/mL; no serious complications; and providing written informed consent before enrollment. The study protocol was approved in all participating institutions.

### Study treatment and dose modification

The study treatment was mFOLFOX6 therapy (L-OHP, 85 mg/m^2^; *1*-LV, 200 mg/m^2^; 5-FU bolus, 400 mg/m^2^; and 5-FU infusion, 2400 mg/m^2^),with a total of 12 courses being administered at 2-week intervals. Further chemotherapy was not performed until recurrence after completion of the scheduled therapy.

If the following criteria for initiation of treatment were not met on the day of or the day before the start of each course, treatment was postponed for a maximum of 29 days: neutrophil count ≥1500/mm^3^, platelet count ≥75,000/mm^3^, and other parameters at the attending physician’s discretion. If adverse events of a high enough grade occurred during the previous course, the dose was reduced by 1 level (up to 2 levels). Bolus 5-FU was discontinued after dose reduction by 1 level, but the dose of *1*-LV was not reduced. If any of the following adverse events occurred (except PSN), the doses of L-OHP and infusional 5-FU were, respectively, reduced to 75 or 55 mg/m^2^ and 1900 or 1400 mg/m^2^: grade ≥3 (or persistent grade 2 for 2 weeks) neutropenia or thrombocytopenia and any other grade 3 non-hematological drug-related adverse events. Infusional 5-FU was reduced to 1900 or 1400 mg/m^2^ if any of the following adverse events occurred: grade 3 (or persistent grade 2 for 2 weeks) diarrhea, oral mucositis, or skin disorders. Study treatment was discontinued if any of the following adverse events occurred: grade 4 diarrhea, oral mucositis, or skin disorders, and other grade 4 non-hematological drug-related adverse events. L-OHP was discontinued if grade ≥3 AR occurred.

The dose of L-OHP was reduced to 75 or 55 mg/m^2^ if the patient developed persistent painless PSN for ≥14 days, painful PSN for 8–13 days, or PSN with dysfunction for ≤7 days. The dose of L-OHP was reduced to 55 mg/m^2^ if the patient developed persistent painful PSN for ≥14 days or PSN with dysfunction for 8–13 days. L-OHP was discontinued if the patient developed persistent PSN with dysfunction for ≥14 days or PSN with dysfunction for 8–13 days following painful PSN for ≥14 days.

### Primary and secondary endpoints

The primary endpoints were the incidence of PSN persisting for ≥8 days that interfered with daily activities and the incidence of grade ≥3 AR. Secondary endpoints were disease-free survival (DFS), relapse-free survival, time to treatment failure, overall survival (OS), adverse events (AEs) including any grade of PSN or AR, comparison of PSN between patients receiving prophylactic therapy with IV calcium/magnesium and/or oral goshajinkigan, etc., at the physician’s discretion and patients not receiving such prophylactic therapy, recovery of PSN during the 3-year follow-up period, the treatment completion rate, the relative dose intensity (RDI), and the number of lymph node metastases, and number of dissected lymph nodes in relation to the prognosis. For prophylactic IV calcium/magnesium, goshajinkigan, and IV calcium/magnesium plus goshajinkigan, cases of concomitant use in the first course were counted.

AEs were evaluated according to the Common Terminology Criteria for Adverse Events (CTCAE) Version 4.0. However, PSN was evaluated by following the NCI-CTC Version 1.0, 2.0 and CTCAE Version 3.0. Dose intensity was calculated as the total dose divided by the duration of dosing, while the planned dose intensity was calculated as the planned dose divided by 14. Then RDI was calculated as dose intensity/planned dose intensity ×100.

### Enrollment and data collection

An electronic data capture system (Viedoc^®^, PCG Solutions, Uppsala, Sweden) was used for registration of the subjects and collection of data related to the endpoints for each patient. Central monitoring was performed, and further information was obtained from the attending physicians as required to confirm the accuracy of the data by each query. The expected enrollment period was 3 years, with an additional follow-up period of 3 years after enrollment of the last patients.

### Sample size and statistical analysis

Assuming that the incidence of grade ≥3 PSN is 12.0 % (threshold incidence: 16.5 %) and the incidence of grade ≥3 AR is 3.0 % (threshold incidence: 6.5 %), based on the results of the MOSAIC trial, when the probability that a two-sided 95 % confidence interval (CI) for the incidence of each adverse event does not contain the threshold incidence is set as 95 %, the number of patients required to evaluate grade ≥3 PSN and AR was calculated to be 798 and 510, respectively. The 95 % CI was calculated by the adjusted Wald-based method [9]. Accordingly, a target of 800 patients was set for enrollment.

The incidence of grade ≥3 PSN and grade ≥3 AR was calculated as percentages with the adjusted Wald-based 95 % CI. If the upper limit of the 95 % CI did not include the threshold incidence, it was concluded that adjuvant mFOLFOX6 therapy was as tolerable for Japanese patients as for Western patients. The Kaplan–Meier method was used to evaluate the relationship between the total dose and the cumulative incidence of PSN or AR. If a drug administration was stopped, data were censored at that point. The median total dose was estimated from the cumulative incidence curve, and the 95 % CI was estimated by the Greenwood formula. In the pre-specified sub-analysis, the influence of prophylactic treatment and other variables on occurrence of grade ≥2 PSN was evaluated in univariate and multivariate logistic regression analysis. Variables evaluated in the model were age, sex, performance status, stage, total dose of L-OHP, and prophylactic pretreatment. SAS Release 9.3 (SAS Institute, Cary, NC, USA) was used for all statistical analyses.

## Results

### Profile of the subjects

**Fig. 1:**
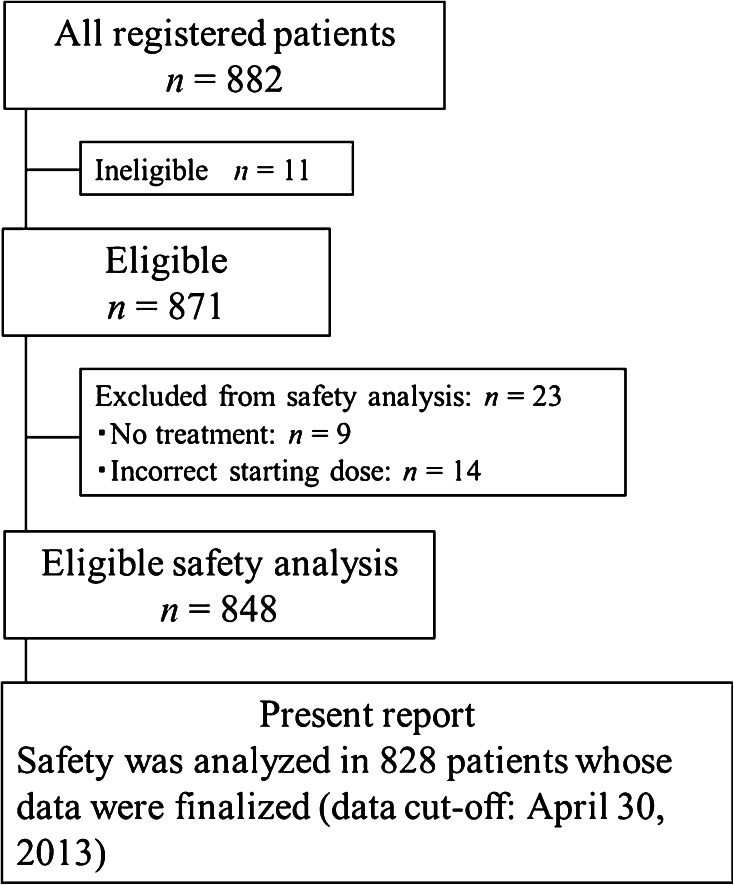
CONSORT diagram

Between November 2010 and March 2012 (17 months), 882 patients were enrolled at 198 institutions. Among these 882 patients, 11 were ineligible, 9 did not start the study treatment, and 14 did not receive the correct initial dosage at their physician’s discretion. These 34 patients were excluded from the safety analysis by central monitoring. Of the remaining 848 patients, 828 patients for whom the treatment status was fixed by April 30, 2013, were included in the safety analysis (Fig. 1). The characteristics of these patients are shown in Table 1.

**Table 1:** Profile of the subjects

	828
Male/female	444/384
Median age (range)	64 (21–83)
Performance status 0/1	776/52
Colon/Rectosigmoid	633/195
Stage^a^ II/IIIa/IIIb	152/402/274
Stage (TNM 7th)^b^ IIA/IIB/IIC/IIIA/IIIB/IIIC	96/33/23/61/437/178
N0/N1/N2/N3^a^	152/402/225/49

^a^General Rules for Clinical and Pathological Studies on Cancer of the Colon, Rectum, and Anus, 7th edition, revised

^b^TNM Classification of Malignant Tumours, 7th edition

### Primary endpoints

The incidence of PSN persisting for ≥8 days that interfered with daily activities and the incidence of grade ≥3 AR were 3.3 % (95 % CI 2.2–4.7) and 1.7 % (95 % CI 1.0–2.8), respectively. At the onset of PSN persisting for ≥8 days that interfered with daily activities, the median total dose of L-OHP and the median number of courses were 672.5 mg/m^2^ and 9, respectively, while the corresponding values were 565.1 mg/m^2^ and 7.5 at the onset of grade ≥3 AR.

### Treatment

The median number of courses was 12 (range: 1–12), and the treatment completion rate was 67.0 %. The median total doses of L-OHP, bolus 5-FU, and infusional 5-FU were 811.1 mg/m^2^, 2798.3 mg/m^2^, and 24,009.0 mg/m^2^, respectively, while the median RDI was 78.2, 87.7, and 78.1 %, respectively.

### Incidence of PSN and AR

**Fig. 2:**
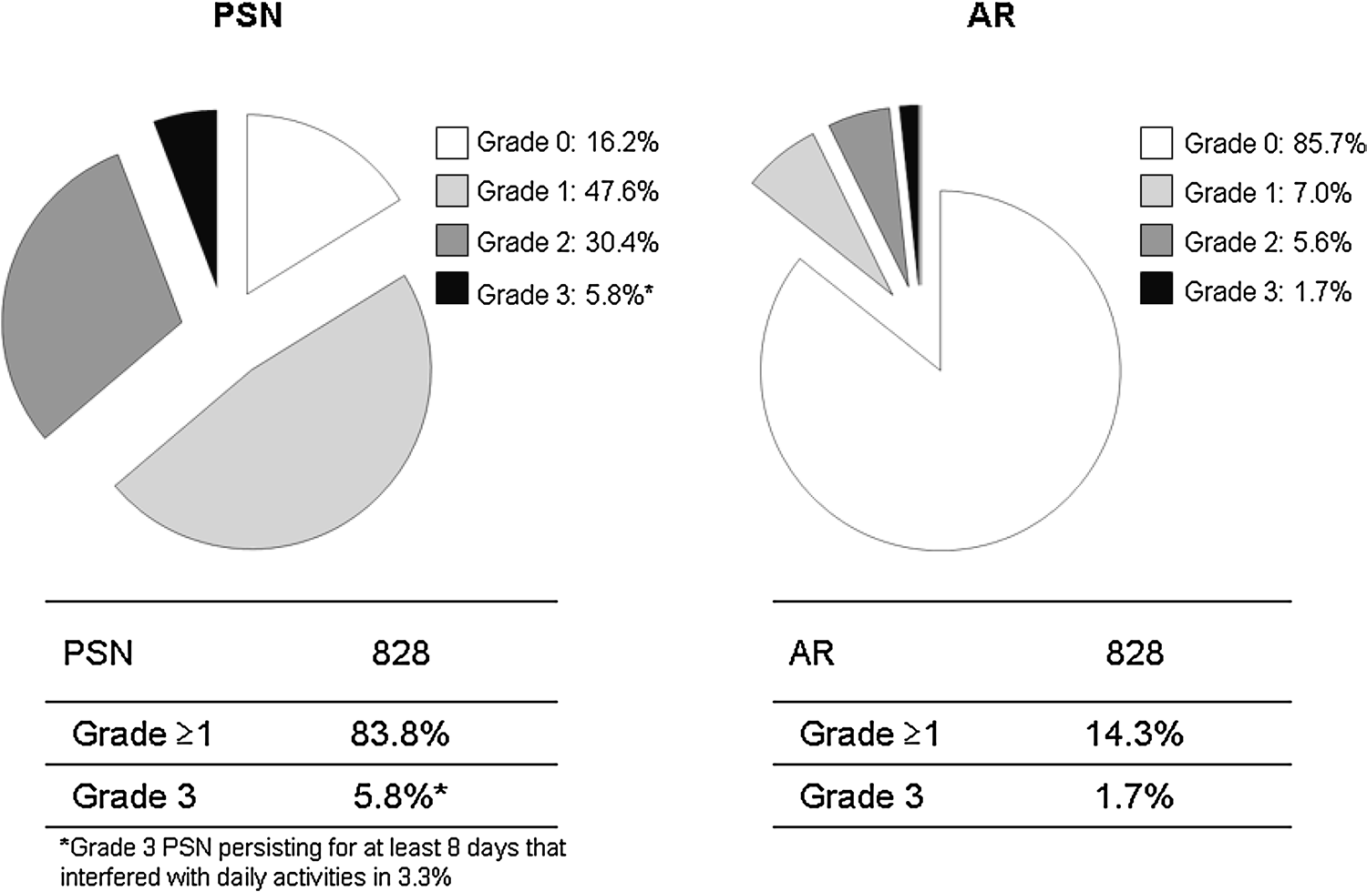
The incidence of PSN and AR

**Fig. 3:**
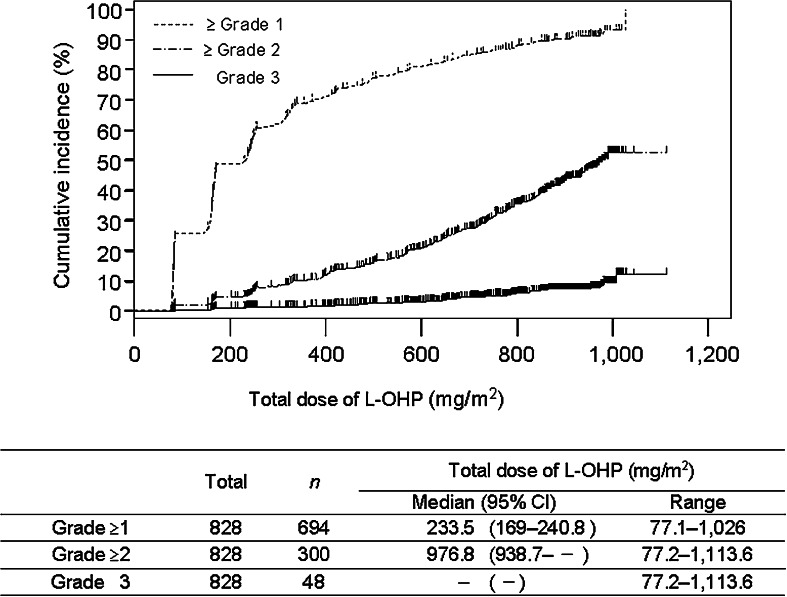
The cumulative incidence of PSN (grade ≥1/grade ≥2/grade 3)

**Fig. 4:**
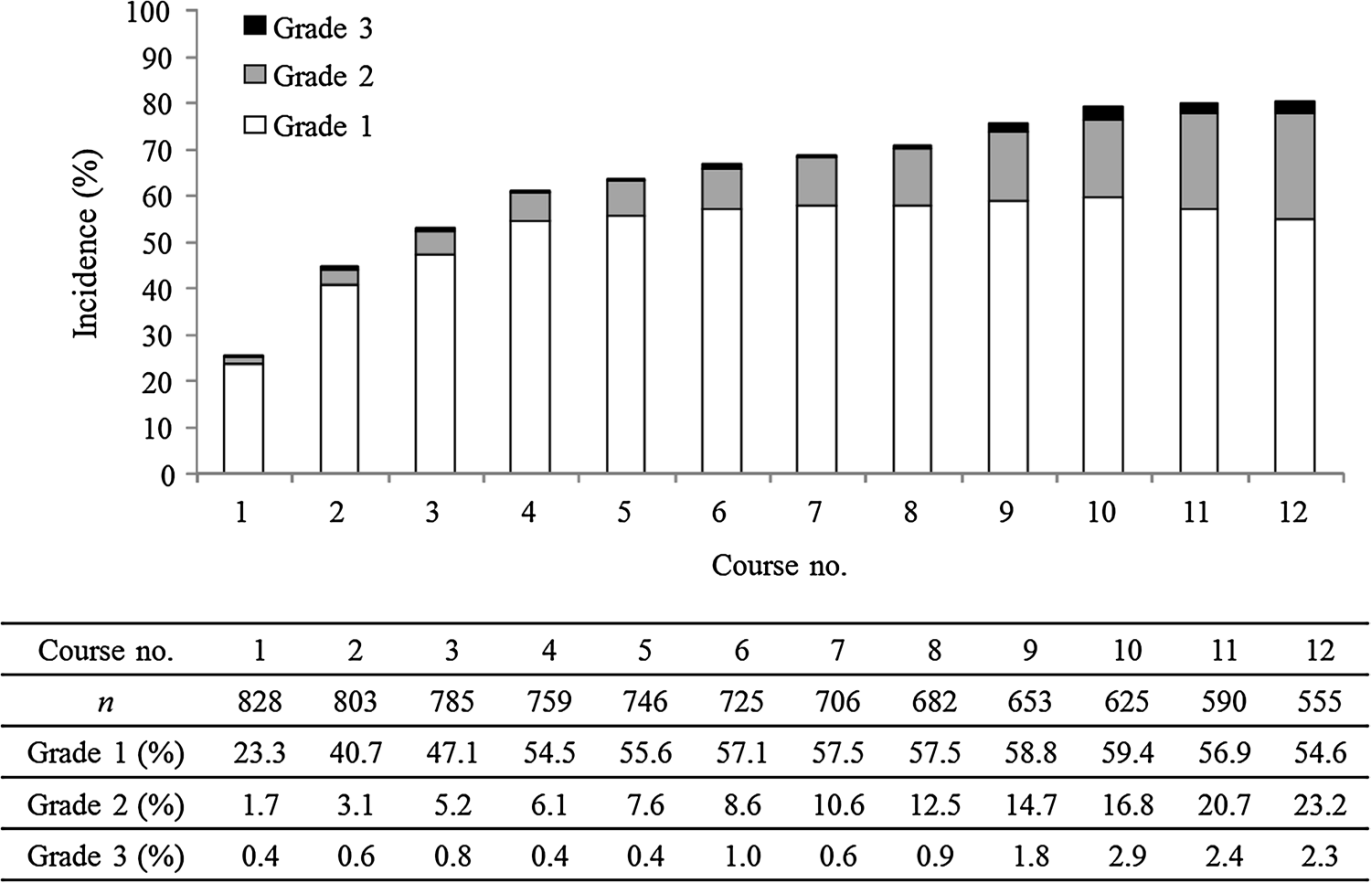
PSN: the incidence of each course (grade 1/grade 2/grade 3)

**Fig. 5:**
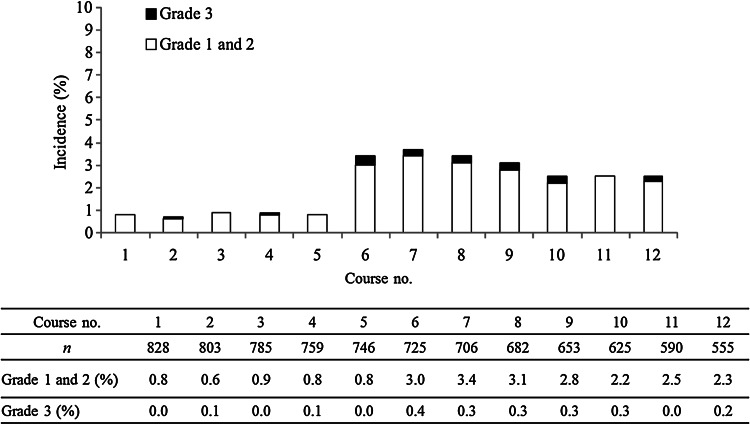
AR: the incidence of each course (grade 1 and 2/grade 3)

The incidence of grade ≥1 PSN was 83.8 %, and the incidence of grade ≥3 PSN was 5.8 %, while the respective values for AR were 14.3 and 1.7 % (Fig. 2). The cumulative incidence of each grade of PSN increased along with the total dose of L-OHP (Fig. 3). While the incidence of grade 1 PSN showed little change after the fourth course, grade 2–3 PSN tended to increase with the course number (Fig. 4). AR was observed from the first course, although its incidence particularly increased after the sixth course (Fig. 5).

### Pre-specified sub-analysis: effect of prophylactic treatment for PSN

Univariate analysis of the factors related to grade ≥2 PSN is shown in Table 2. In addition to a total dose of L-OHP ≥510 mg/m^2^, there was a significant difference in relation to the use of calcium/magnesium and use of goshajinkigan as prophylactic treatment at the initial course of mFOLFOX6, showing the likelihood of detrimental effects on grade ≥2 PSN. Table 3 shows the results of the multivariate analysis of factors related to the occurrence of grade ≥2 PSN, with a significant difference being observed only for a total L-OHP dose of ≥510 mg/m^2^. The odds ratios for prophylactic monotherapy with calcium/magnesium, monotherapy with goshajinkigan, and concomitant therapy with both agents were 1.345, 1.624, and 2.184, respectively.

**Table 2:** Relationship of variables with the onset of PSN of grade ≥2: univariate analysis

Explanatory variables	Logistic regression analysis			
	*n*	Odds ratio Point estimate	95 % confidence interval	*P* value
Age				
<70 years old	588	1.000	–	–
≥70 years old	240	0.794	0.579–1.090	0.1539
Sex				
Male	444	1.000	–	–
Female	384	1.256	0.946–1.669	0.1153
Performance status				
0	776	1.000	–	–
1	52	1.314	0.743–2.321	0.3477
Stage				
II	152	1.000	–	–
III	676	1.114	0.770–1.613	0.5662
Total dose of L-OHP				
<510 mg/m^2^	158	1.000	–	–
≥510 mg/m^2^	670	2.113	1.417–3.151	<0.001
Prophylactic pretreatment				
Without Ca/Mg	696	1.000	–	–
With Ca/Mg	132	1.583	1.087–2.307	0.0168
Without goshajinkigan	733	1.000	–	–
With goshajinkigan	95	2.045	1.330–3.145	0.0011

For prophylactic Ca/Mg and goshajinkigan, cases of concomitant use in the first course were counted

**Table 3:** Relationship of variables with the onset of PSN of grade ≥2: Multivariate analysis

Explanatory variables	Logistic regression analysis			
	*n*	Odds ratio Point estimate	95 % confidence interval	*P* value
Age				
<70 years old		1.000	–	–
≥70 years old		0.780	0.562–1.083	0.1377
Sex				
Male		1.000	–	–
Female		1.266	0.944–1.697	0.1155
Performance status				
0		1.000	–	–
1		1.287	0.714–2.319	0.4021
Stage				
II		1.000	–	–
III		0.999	0.683–1.461	0.9944
Total dose of L-OHP				
<510 mg/m^2^		1.000	–	–
≥510 mg/m^2^		2.132	1.420–3.201	<0.001
Prophylactic pretreatment				
No prior treatment	627	1.000	–	–
Ca/Mg (monotherapy)	106	1.345	0.808–2.238	0.2573
Goshajinkigan (monotherapy)	69	1.624	0.977–2.699	0.0612
Ca/Mg + goshajinkigan (concomitant therapy)	26	2.184	0.975–4.895	0.0577

For prophylactic Ca/Mg, goshajinkigan, and Ca/Mg plus goshajinkigan, cases of concomitant use in the first course were counted

### Adverse events

The AEs that occurred during this study are shown in Table 4. The only grade ≥3 AE occurring in more than 10 % of the patients was neutropenia, which was observed in 28.7 %. However, the incidence of febrile neutropenia was 0.4 %. There was only one case of grade 3 interstitial pneumonitis, and there were no treatment-related deaths.

**Table 4:** Adverse events

	*n* (%)	
	Grade ≥1	Grade ≥3
Neutropenia	441 (53.3 %)	238 (28.7 %)
Leucopenia	265 (32.0 %)	21 (2.5 %)
Thrombocytopenia	264 (31.9 %)	14 (1.7 %)
Diarrhea	153 (18.5 %)	17 (2.1 %)
Anorexia	255 (30.8 %)	17 (2.1 %)
Nausea	311 (37.6 %)	14 (1.7 %)
ALT (GPT) increased	199 (24.0 %)	13 (1.6 %)
Fatigue	205 (24.8 %)	10 (1.2 %)
Vomiting	90 (10.9 %)	6 (0.7 %)
AST (GOT) increased	211 (25.5 %)	5 (0.6 %)
Febrile neutropenia	3 (0.4 %)	3 (0.4 %)
Pneumonitis (interstitial pneumonitis)	7 (0.8 %)	1 (0.1 %)

CTCAE version 4.0

## Discussion

To our knowledge, this is the first large-scale study of PSN persisting for ≥8 days that interfered with daily activities and grade ≥3 AR in Asian patients receiving mFOLFOX6 as adjuvant chemotherapy after curative resection of stage II or III colon cancer. The incidence of persistent PSN was 3.3 % (95 % CI 2.2–4.7), while that of grade ≥3 AR was 1.7 % (95 % CI 1.0–2.8). Since the upper limit of the 95 % CI of the incidence of each event (PSN: 4.7 %; AR: 2.8 %) was lower than the threshold incidence (PSN: 16.5 %; AR: 6.5 %), the primary endpoint was met and we confirmed that both persistent PSN and grade ≥3 AR were not statistically more frequent than the reported incidences in Western patients during the MOSAIC and NSABP C-08 trials.

We selected mFOLFOX6 as the regimen for this study because it is more simple and easier than FOLFOX4 in terms of administration procedure. mFOLFOX6 is widely used around the world (for example, it was the reference regimen in the NSABP C-08 study [5, 6]) because a cross-sectional comparison of these two regimens for advanced and/or recurrent colorectal cancer showed comparable efficacy and safety [10].

Table 5 displays a comparison of the present findings with the results of the MOSAIC [3], MASCOT [7], and NSABP C-08 [5] trials regarding safety and administration. In the present study, the incidence of grade ≥3 PSN was 5.8 % (3.3 % for PSN persisting for ≥8 days that interfered with daily activities), which was lower than the incidence in the MOSAIC trial (12.4 %) and the NSABP C-08 trial (14.4 %), and comparable with that in the MASCOT trial (5.7 %). The incidence of grade ≥3 AR was 1.7 % in this study, which was lower than in the MOSAIC (2.9 %), MASCOT (3.1 %), or NSABP C-08 (4.7 %) trials. Comparison between this study and the MOSAIC trial showed that the median course number was 12 in both, while the cumulative treatment completion rate was 67.0 % and 74.7 %, the median total dose of L-OHP was 811.1 and 810.0 mg/m^2^, and the median RDI for L-OHP was 78.2 and 80.5 %, respectively. In relation to tolerability, it is worth noting that the administration of therapy was similar in both studies, but the incidence of grade ≥3 PSN and grade ≥3 AR was lower in the present study. This was probably because it was possible to reduce the L-OHP dose by up to two levels in the present study depending on the severity and duration of PSN.

**Table 5:** Comparison among the JOIN, MOSAIC, MASCOT, and NSABP C-08 trials

	JOIN (*n* = 828)	MOSAIC (*n* = 1123)	MASCOT (*n* = 159)	NSABP C-08 (*n* = 1350)
Median number of courses	12	12	12	–
Treatment completion rate (%)	67.0	74.7	81.8	–
Median total dose of L-OHP (mg/m^2^)	811.1	810.0	–	850.0
Median RDI of L-OHP (%)	78.2	80.5	84.0^a^	95.4
Adverse events (grade ≥ 3)				
Neutropenia (%)	28.7	41.0^c^	52.2	32.6^d^
PSN (%)	5.8 (3.3^b^)	12.4^c^	5.7	14.4^d^
AR (%)	1.7	2.9^c^	3.1	4.7^d^

^a^Calculated from the dose intensity described in the report

^b^PSN persisting for at least 8 days that interfered with daily activities

^c^Based on the data for 1.108 subjects

^d^Based on the data for 1321 subjects

The sub-analysis did not demonstrate efficacy of prophylactic treatment for PSN. In a previous phase III clinical study [11], calcium/magnesium adjuvant therapy did not show efficacy against PSN induced by L-OHP and the present findings were consistent with that report. In a previous phase II randomized controlled study performed in patients with advanced/recurrent colorectal cancer, the Kampo medicine goshajinkigan was reported to be effective against PSN induced by FOLFOX4 therapy or mFOLFOX6 therapy [12]. However, our multivariate analysis showed that prophylactic monotherapy with calcium/magnesium, goshajinkigan, or concomitant therapy with calcium/magnesium and goshajinkigan did not prevent the development of grade ≥2 PSN [hazard ratio (HR) 1.345, 95 % CI 0.808–2.238; HR 1.624, 95 % CI 0.977–2.699; HR 2.184, 95 % CI 0.975–4.895, respectively]. Similar to our study, a phase III randomized double-blind clinical study was conducted using goshajinkigan as adjuvant therapy [13]. At the interim analysis, efficacy for PSN induced by mFOLFOX6 therapy was not demonstrated and the study was terminated prematurely. In our study, prophylactic treatment with calcium/magnesium or goshajinkigan was selected at the discretion of the attending physician. Therefore, we must be cautious about potential bias as a result of non-randomized selection of patients among specific study sites and detailed explanations to patients about PSN from the investigator. Also, a relatively small number of patients on prophylactic treatment were studied, although our current results were obtained from a pre-specified sub-analysis. However, there have been no other reports about prophylactic treatment with concomitant calcium/magnesium and goshajinkigan. We found that even combining both agents did not have a preventive effect against PSN induced by L-OHP. If a prospective investigation of prophylaxis for PSN is conducted in the future, our current findings should be taken into considerations when developing the study design.

The incidence of grade ≥3 neutropenia was 28.7 % in this study, which was lower than in the MOSAIC (41.0 %) and MASCOT (52.2 %) trials. This was probably related to the difference in the number of bolus 5-FU doses between mFOLFOX6 therapy used in the present study and FOLFOX4 therapy used in the MOSAIC and MASCOT trials. In the NSABP C-08 study [5], mFOLFOX6 was administered as adjuvant chemotherapy to patients with curatively resected stage II or III colon cancer and the incidence of grade ≥3 neutropenia was 32.6 %, which was comparable with that in the present study.

Among the 828 patients analyzed in this study, about 20 % had stage II disease, about 60 % were in stage IIIA or IIIB, and about 20 % were in stage IIIC. The clinical usefulness of adjuvant chemotherapy for stage II colon cancer remains controversial because some reports have supported its efficacy [14–18] and others have not [19, 20]. Therefore, it is interesting that stage II patients accounted for approximately 20 % of our subjects. This may be because exploratory analysis of 569 patients with stage II colon cancer from the MOSAIC trial [4] (282 in the FOLFOX4 group and 287 in the LV5FU2 group) who had risk factors for recurrence (one or more of T4, tumor perforation, bowel obstruction, undifferentiated tumor, vascular invasion, or < 10 lymph nodes examined) showed that DFS at 5 years was better in the FOLFOX4 group than in the LV5FU2 group [82.1 vs. 74.9 %, HR 0.74 (95 % CI 0.52–1.06)].

The median age of the 828 patients analyzed in our study was 64 years, and 240 patients were aged 70 years or older (29.0 %). In contrast, the eligibility criteria of the MOSAIC trial included an age under 76 years, and only 315 out of 2246 patients were aged 70 years or older (14.0 %) [21]. Presumably, patients aged 70 years or older accounted for 29.0 % of the present study population because an upper age limit was not set in the eligibility criteria and because the JSCCR Guidelines 2010 for the Treatment of Colorectal Cancer [2] also recommend adjuvant chemotherapy for elderly patients aged ≥70 years, depending on their PS and organ function. Results for the secondary endpoints (efficacy including DFS and the long-term outcome of PSN) are not reported here and will be reported in the future.

Currently, the International Duration Evaluation of Adjuvant Chemotherapy (IDEA) collaboration is investigating whether the duration of L-OHP-based adjuvant chemotherapy can be reduced from 6 to 3 months while maintaining efficacy, and pooled analysis will be performed using data from multiple large-scale clinical studies that are ongoing around the world [22]. In Japan, the JFMC47-1202-C3 study of stage III colon cancer and the JFMC48-1301-C4 study of stage II colon cancer with risk factors for recurrence are participating in IDEA.

In conclusion, this study showed that adjuvant mFOLFOX6 therapy is tolerated in Japanese patients with curatively resected stage II or III colon cancer.
